# Technical tips and procedural steps in endovascular aortic aneurysm repair with concomitant recanalization of iliac artery occlusions

**DOI:** 10.1186/2193-1801-2-605

**Published:** 2013-11-13

**Authors:** Jorge Senkichi Uchiyamada, Shigeo Ichihashi, Shinichi Iwakoshi, Hirofumi Itoh, Nobuoki Tabayashi, Kimihiko Kichikawa

**Affiliations:** Department of Radiology, Nara Medical University, 840 Shijo-cho, Kashihara, Nara, 634-8521 Japan; Department of Thoracic and Cardiovascular Surgery, Nara Medical University, 840 Shijo-cho, Kashihara, Nara, 634-8521 Japan

**Keywords:** Abdominal aortic aneurysm, Endovascular aneurysm repair, Iliac artery occlusion, Peripheral arterial disease, Stent placement

## Abstract

**Purpose:**

The goal of this paper is to describe our technical strategy and procedural steps for endovascular aneurysm repair (EVAR) when performed with concomitant recanalization of the iliac artery occlusion.

**Materials and methods:**

Three octogenarians having abdominal aortic aneurysm (AAA)/common iliac artery aneurysms (CIAA) with unilateral external iliac artery (EIA) occlusion underwent EVAR with recanalization of the occluded iliac arteries. Crossing the iliac artery occlusions was attempted in a retrograde approach using a 0.035 inch-hydrophilic guidewire. After passage of a guidewire, predilation was performed using 6mm balloon. Then a 12-Fr sheath was advanced via the occluded EIA for contralateral iliac limb delivery. Internal iliac artery embolization was subsequently performed as needed. A self-expanding stent was then placed in the occluded EIA after EVAR.

**Results:**

Recanalization of the EIA occlusion, followed by stentgraft delivery through the occlusion and EVAR, was successfully performed in all three patients. Penetration of the occluded EIA was successfully achieved only by retrograde approach in two patients, and by bidirectional approach in the other patient. No perioperative complication or death occurred. Postoperative CT showed no type I or III endoleaks in the aneurysms and patent iliac arteries in all patients.

**Conclusions:**

Total endovascular repairs were successfully performed for three patients with AAA and concomitant unilateral EIA occlusions. The proposed steps described in this report might reduce the complication rate and enhance the technical success rate associated with this procedure.

## Introduction

Endovascular aneurysm repair (EVAR) is less invasive and is associated with a lower 30-day mortality rate when compared with open surgery. As a result, its use has increased worldwide (Greenhalgh et al. 2010, De Bruin et al. 2010). However, the feasibility and outcomes of EVAR are highly dependent on various anatomic factors, including the stent-graft access route of the iliac artery. The reported incidence of peripheral arterial disease (PAD) with concomitant abdominal aortic aneurysm (AAA) ranges between 10% and 40% (Wanhainen et al. 2005). Iliac recanalization of occlusive disease is commonly performed during management of aorto-iliac occlusive disease (AIOD), but only few reports have described this technique combined with bifurcated EVAR (Vallabhaneni et al. 2012Scurr et al. 2007). Further, no reports have described the technical strategy and procedural steps needed to achieve successful results without complications. Therefore, the purpose of this report was to describe our experiences in regards to three cases of EVAR after recanalization of occluded iliac arteries, focusing on our technical strategy and procedural steps.

## Materials and methods

EVAR with concomitant recanalization of unilateral external iliac artery (EIA) occlusion was performed in three octogenarians. All patients had AAA, and two patients had concomitant common iliac artery (CIA) aneurysms, one of which was present ipsilateral to the EIA occlusion. All procedures were performed via the bilateral common femoral arteries under local anesthesia with conscious sedation. An Excluder endograft (W. L. Gore and Associates, Flagstaff, AZ, USA) was chosen for its simple maneuver and lowest delivery profile of the contra-lateral leg at the time of procedures. After cut down of the bilateral common femoral arteries, 8-Fr sheaths were inserted into the bilateral common femoral arteries. Passage of the iliac artery occlusions was attempted in a retrograde manner using a 0.035-inch hydrophilic guidewire (Radifocus, Terumo, Tokyo, Japan). When this failed, a bidirectional approach with a 10mm snare kit (Amplatz GooseNeck Snare kit, ev3 Endovascular, Inc. Plymouth, MN, USA) was performed to establish a through and through wire. If recanalization of the occluded iliac artery was successfully achieved, predilation was performed using a 6mm sized balloon. Then, a 12-Fr sheath (Dryseal sheath, W. L. Gore and Associates, Flagstaff, AZ, USA) was advanced via the occluded iliac artery for delivery of a contralateral iliac limb, which required a lower profile sheath when compared with the 18-Fr sheath of the main body device. Unilateral IIA embolization was thereafter performed in patients who required stent-graft extension to the EIA for treatment of concomitant CIA aneurysms. After deployment of the stent-graft in a routine manner and confirmation of the absence of major endoleaks, a self-expanding E-Luminexx vascular stent (Bard Peripheral Vascular, Inc., Tempe, AZ, USA) was placed in the occluded EIA. This study was performed according to the guidance of Helsinki Declaration. All patients signed an informed consent form regarding procedures.

## Results

**Figure 1:**
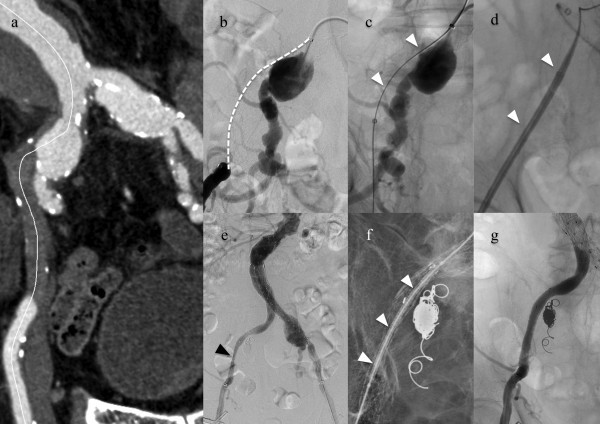
**An example of the procedural steps.** (Case 1), **a.** Curved multiplanar reconstruction of the CTA showed AAA, bilateral CIAA and right EIA occlusion. **b.** Angiogram showed CTO of the right EIA (dashed line) and right CIAA. **c.** Lesion was successfully crossed using bidirectional wiring (white arrow head). **d.** After predilation, a 14-Fr sheath was advanced along the wire (white arrow head). **e.** Completion angiogram showed residual stenosis at the right EIA despite placement of a self-expanding stent (black arrow head). **f.** Express LD stent was deployed to support the self-expanding stent (white arrow head). **g.** Successful dilation of the EIA was achieved. AAA: abdominal aortic aneurysm, CIAA: common iliac artery aneurysm, CTO: chronic total occlusion, EIA: external iliac artery.

Recanalization of the occluded EIA and implantation of the Excluder endograft was successfully performed in all cases. In one patient with concomitant bilateral CIA aneurysms (Case 1), the large leg of an Endurant endograft (ETEW 2828C82E; Medtronic Cardiovascular, Santa Rosa, CA, USA) was deployed in the left CIA to avoid bilateral internal iliac artery (IIA) embolization that could otherwise cause bowel ischemia or buttock claudication. Penetration of the occluded EIA was successfully achieved only by retrograde approach in two patients. However, in one patient (Case 1), the retrograde guidewire ran through the sub-intimal space and could not get re-entry to the true lumen, and bidirectional approach was subsequently employed. Unilateral IIA embolization was then performed in two patients (Case 1, 3) who required stent-graft extension to the EIA for treatment of concomitant CIA aneurysms. In one case of sub-intimal recanalization (Case 1), insufficient expansion of the E-Luminexx stent was recognized, requiring placement of a balloon-expandable stent Express LD (Boston Scientific Corporation, Natick, MA, USA) in a stent-in-stent manner to support stent dilation (Figure 1). All three patients were returned to the general ward without requiring intensive care, and ambulation was allowed beginning on the next morning. No major adverse event or death occurred. Contrast-enhanced CT was performed 1 week after the procedures, confirming exclusion of the aneurysms and patency of the iliac stent. Perioperative and postoperative parameters are summarized in Table 1.

**Table 1:** **Patient characteristics**

	Case 1	Case 2	Case 3
Gender	Male	Male	Male
Age	86 years old	92 years old	84 years old
Comorbidity	Alzheimer	Hypertension	Hypertension, CKD, CVD
Aneurysm diameter	AAA 53 mm	AAA 56 mm	AAA 36 mm
	R-CIAA 25mm	L-IIAA 26 mm	R-CIAA 56 mm
	L-CIAA 25mm		
Occluded EIA			
-Side	R	R	L
-Length/ Diameter	85 mm/8 mm	75 mm/9 mm	6 mm/7 mm
Recanalization	Bidirectional	Retrograde	Retrograde
IIA embolization	R	-	R
Landing site (R/ L)	EIA/ CIA	CIA/ CIA	EIA/ CIA
Device size	Mainbody: 28.5mm/12mm/12cm	Mainbody: 26mm/12mm/14cm	Mainbody:22mm/12mm/18cm
	Rt leg: 12mm/14cm,	Rt leg: 14.5mm/14cm,	Rt leg: 12mm/10cm, 10mm/7cm
	Lt leg (Endurant): 28mm/9.3cm	Lt leg: 18mm/11.5cm	Lt leg: 18mm/13.5cm
Complication			
-During EVAR	No	No	No
-Follow-up (CT/MR)	Type II Endoleak	Type II Endoleak	Type II Endoleak
ABI at occluded ilac site			
pre/ post- treatment	0.5/ 0.93	0.72/ 0.91	0.62/ 0.61
Rutherford classification at occluded iliac site			
Pre/ post- treatment	2/ 0	2/ 0	0/ 0
Aneurysm diameter	AAA 47 mm	AAA 52 mm	NA
(after 6 months)	R-CIAA 23mm	L-IIAA 27 mm	
	L-CIAA 25mm		

ABI; ankle-brachial index, CIAA; common iliac artery aneurysm, CKD; chronic kidney disease; CVD; cerebro vascular disease, EIA; external iliac artery aneurysm, EVAR; endovascular aneurysm repair, IIAA; internal iliac artery aneurysms, L; left, NA; not available, R; right.

## Discussion

AAA with concomitant AIOD is not uncommon, and 15.4% of patients were excluded from EVAR because of inadequate access vessel size for stent-graft delivery (Joels et al. 2009). Open surgery is the traditional treatments for patients with aortic aneurysms and AIOD, but compared with endovascular treatment, this strategy is associated with higher rates of perioperative morbidity and mortality in the elderly or obese patients, and is technically challenging on patients with hostile abdomen due to previous open surgery or radiation (Oderich et al. 2012). Hybrid strategies using iliac conduits also have been used for endovascular repair of aneurysms in patients with poor vascular access, but conduit construction requires retroperitoneal incision, which is also associated with higher complication rates and longer hospital stay when compared with total endovascular procedures (Lee et al. 2003). An alternative strategy is the use of aortouniiliac (AUI) stent-graft repair with crossover femoro-femoral bypass graft, however, compared with bifurcated stent grafts, decreased patency and higher risk of perioperative complications such as femoral graft infections were reported (Clouse et al. 2003).

Recent advancements in medical devices and technical expertise have allowed the performance of total endovascular repair for patients with complex aorto-iliac pathologies. Yano et al. (2001), Hinchliffe et al. (2007), and Peterson and Matsumura (2008) described the usefulness of ancillary technique such as PTA and endoluminal conduit to facilitate endograft delivery through the stenosed iliac arteries. Regarding AAA with iliac artery occlusions, Vallabhaneni et al. (2012) reported a technical success rate of 93.3% when using endovascular recanalization. Our present report also describes successful performance of EVAR after recanalization of occluded iliac arteries without major complication in all three octogenarian patients. Recanalization of the occluded iliac artery and use of a bifurcated device allows restoration of arterial flow with low risk of infection and a high rate of patency.

The ability to perform this procedure depends on recanalization of the occluded artery and the ability to advance the delivery sheath. We successfully crossed the lesion using a 0.035-inch guidewire in two patients using a retrograde approach and in one patient using bidirectional wiring from the ipsilateral and contralateral iliac arteries. Previous reports regarding primary stent placement for iliac artery occlusive disease showed a high rate of technical success for crossing the iliac artery occlusions when using bidirectional approach (Ichihashi et al. 2011). In the case of the EIA occlusion coexisting the CIA aneurysm ipsilaterally like Case 1 (Figure 1), there could be a risk of aneurysm wall perforated by the guidewire when the guidewire run through the subintimal space of the aneurysm wall, which could be fragile compared to the wall of the non-aneurysmal part. Careful handling of the guidewire is required. When the guidewire from the retrograde approach can not re-enter into the true lumen, using re-entry device could be one option for avoiding the excessive manipulation in the subintimal lumen of the aneurysms. To date re-entry devices are not available in Japan.

We performed predilation of the occluded iliac artery using 6mm balloons, because outer diameter of the 12F sheath is around 5mm. Overdilation of iliac arteries could increase the risk of arterial rupture, especially in the case of diffuse calcified iliac arteries. In this regard, it is reasonable to choose the occluded iliac side as a pathway for the contralateral limb. If severe calcification is identified by CT preoperatively, internal endoconduit using stentgraft followed by non-compliant balloon dilation could be useful for avoiding arterial ruptures (Hinchliffe et al. 2007 and Peterson and Matsumura 2008). However this technique may require the coverage of origin of the internal iliac artery, which could cause the spinal cord ischemia in the patient treated for concomitant thoracic or thoracoabdominal aneurysms. In such cases, open surgical endoconduit could decrease the risk by preservation or reconstruction of the internal iliac artery (Oderich et al. 2012).

IIA embolization was performed after the successful advancement of a sheath through the occluded iliac artery. If IIA embolization (especially at the same side of the occluded EIA) was performed before recanalization of the occluded artery, blood flow to the lower extremity via pelvic collateral channels might be profoundly decreased for a prolonged time, resulting in severe ischemia and muscle necrosis, because we could not predict the required time for penetration of the occlusion in contrast to the stenosis.

Stent placement for an occluded artery was routinely performed after EVAR in the present cases. Previous studies suggested that primary stenting produced a better patency rate when compared with balloon angioplasty with provisional stenting when used for iliac artery occlusion (Ichihashi et al. 2011). Stent placement in the iliac artery prior to EVAR may interfere the passage of the sheath or touch-up balloon. Lam et al. (2008) described a case of migrated self-expanding stent after endograft passage. Whether complications during the stentgraft delivery, such as stent migration or stent collapse occur or not depends on the degree of the expansion of the stent itself. If the stent expands insufficiently due to the arterial recoil or calcification, the risk of complications could become higher. Except when using the technique of internal endoconduit, we believe the stent placement should be performed after EVAR to lower the complication risk.

In conclusion, totally endovascular repair was successfully performed for three patients with AAA and concomitant unilateral EIA occlusions. The proposed steps described in this report might reduce the complication rate and enhance the technical success rate associated with this procedure.
